# Different FDG‐PET metabolic patterns of anti‐AMPAR and anti‐NMDAR encephalitis: Case report and literature review

**DOI:** 10.1002/brb3.1540

**Published:** 2020-01-27

**Authors:** Yi‐Chia Wei, Jing‐Ren Tseng, Chia‐Lun Wu, Feng‐Chieh Su, Wei‐Chieh Weng, Chih‐Chin Hsu, Kai‐Hsiang Chang, Chun‐Feng Wu, Ing‐Tsung Hsiao, Ching‐Po Lin

**Affiliations:** ^1^ Department of Neurology Chang Gung Memorial Hospital Keelung Taiwan; ^2^ Institute of Neuroscience National Yang Ming University Taipei Taiwan; ^3^ Community Medicine Research Center Chang Gung Memorial Hospital Keelung Taiwan; ^4^ Collage of Medicine Chang Gung University Taoyuan Taiwan; ^5^ Nuclear Medicine and Molecular Imaging Center Chang Gung Memorial Hospital Linkou Taiwan; ^6^ Department of Medical Imaging and Radiological Sciences College of Medicine Chang Gung University Taoyuan Taiwan; ^7^ Department of Hematology and Oncology Chang Gung Memorial Hospital Keelung Taiwan

**Keywords:** autoimmune encephalitis, FDG‐PET, hypermetabolism, hypometabolism, receptor density map

## Abstract

**Introduction:**

^18^F‐fluorodeoxyglucose (FDG)‐PET metabolic patterns of brain differ among autoimmune encephalitis with different neuronal surface antigens. In this case report, we compared the topographical relationship of cerebral glucose metabolism and antigen distribution in the patients with anti‐NMDAR and anti‐AMPAR encephalitis. Literature review summarized the common features of brain metabolism of autoimmune encephalitis.

**Methods:**

The cerebral glucose metabolism was evaluated by FDG‐PET/CT during acute‐to‐subacute stage of autoimmune encephalitis and after treatment. The stereo and quantitative analysis of cerebral metabolism used standardized *z*‐score and visualized on three‐dimensional stereotactic surface projection. To map NMDAR and AMPAR in human brain, we adopted genetic atlases from the Allen Institute and protein atlases from Zilles's receptor densities.

**Results:**

The three‐dimensional stereotactic surface projection displayed frontal‐dominant hypometabolism in a 66‐year‐old female patient with anti‐AMPAR encephalitis and occipital‐dominant hypometabolism in a 29‐year‐old female patient with anti‐NMDAR encephalitis. Receptor density maps revealed opposite frontal–occipital gradients of AMPAR and NMDAR, which reflect reduced metabolism in the correspondent encephalitis. FDG‐PET hypometabolic areas possibly represent receptor hypofunction with spatial correspondence to receptor distributions of the autoimmune encephalitis. The reversibility of hypometabolism was in line with patients' cognitive improvement. The literature review summarized six features of metabolic anomalies of autoimmune encephalitis: (a) temporal hypermetabolism, (b) frontal hypermetabolism and (c) occipital hypometabolism in anti‐NMDAR encephalitis, (d) hypometabolism in association cortices, (e) sparing of unimodal primary motor cortex, and (e) reversibility in recovery.

**Conclusions:**

The distinct cerebral hypometabolic patterns of autoimmune encephalitis were representative for receptor hypofunction and topographical distribution of antigenic receptors. The reversibility of hypometabolism marked the clinical recovery of autoimmune encephalitis and made FDG‐PET of brain a valuable diagnostic tool.

## Graphical abstract

Glucose metabolism patterns were different in anti‐AMPAR and anti‐NMDAR encephalitis. Hypometabolism was frontal‐dominant in anti‐AMPAR encephalitis but occipital‐dominant in anti‐NMDAR encephalitis. The topographical differences might be related to receptor density.

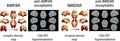

## 1 INTRODUCTION

Except for mesial temporal hyperintensity or whole‐brain atrophy, structural brain images are usually nonspecific in autoimmune encephalitis (Wei et al., 2013). ^18^F‐fluorodeoxyglucose positron emission tomography and computed tomography (FDG‐PET/CT) evaluates the glucose metabolism of the brain and has been used to assess inflammatory and infectious diseases (Jamar et al., 2013; Tseng et al., 2013), but its application in evaluating immune‐mediated encephalitis is emerging too. The diagnostic criteria reported by Graus et al indicated the role of FDG‐PET in the detection of definite autoimmune limbic encephalitis by bilateral mesial temporal signal abnormalities in FDG‐PET (Graus et al., 2016). Sensitivity was higher in FDG‐PET than in MRI, because mesial temporal hypermetabolism can be identified in patients with normal MRI (Graus et al., 2016). Glucose hypometabolism has also been noticed in cortices that appeared normal on the MRI of patients with autoimmune encephalitis (Baumgartner, Rauer, Mader, & Meyer, 2013; Heine et al., 2015).

In this case report and literature review, we evaluated the cerebral glucose metabolism of patients with autoimmune encephalitis at the acute‐to‐subacute stages and after treatment. We used standardized statistics and spatial visualization to compare tests between patients. To better understand the differences of metabolism in patients with distinct autoantibodies, we adopted genetic and protein atlases of the human brain as references.

## 2 METHODS

### 2.1 Study design

We report two cases of autoimmune encephalitis during 2018–2019 at the Chang Gung Memorial Hospital in Keelung (Taiwan). The patients with a clinical diagnosis that fulfilled the Graus criteria of possible autoimmune encephalitis were enrolled and signed an informed consent form (Graus et al., 2016). This study was approved by the Institutional Review Board of Chang Gung Memorial Hospital (approval number 201700701A3).

### 2.2 Antibody detection

Autoantibodies were identified through a cell‐based assay using an indirect immunofluorescence test (EUROIMMUN, Germany). Antigens transfected onto HEK293 cells were N‐methyl‐D‐aspartate receptor (NMDAR), α‐amino‐3‐hydroxy‐5‐methyl‐4‐isoxazolepropionic acid receptor (AMPAR), contactin‐associated protein‐like 2, leucine‐rich glioma‐inactivated protein 1, gamma‐aminobutyric acid B receptor, and dipeptidyl‐peptidase‐like protein‐6. After incubating the HEK293 cells with patients' serum, plasma, or CSF, fluorescein‐labeled anti‐human IgG (goat) was used as conjugate. The green fluorescence of the fluorescein could be observed when excited using a laser with an excitation filter 450–490 nm and represented the existence of autoantibodies.

### 2.3 FDG‐PET/CT scan

All of the patients received brain FDG‐PET/CT scans when they regained consciousness and were able to undergo scanning. The duration from onset to the first PET scan depended on individual disease severity. After recovery to an independent state (modified Rankin Scale [mRS] score 1–2), patients underwent a follow‐up PET scan. All FDG‐PET/CT scans were performed using a Biograph mCT PET/CT system (Siemens Healthineers) in a three‐dimensional acquisition mode. A 10‐min PET scan was acquired starting approximately 30 min after the injection of 185 ± 18 MBq of FDG. All PET images were reconstructed using the 3D‐ordered subset expectation maximization (OSEM) algorithm (4 iterations, 24 subsets; Gaussian filter 2 mm, zoom 3) with CT‐based attenuation correction as well as scatter and random corrections. The reconstructed images had a matrix size of 400 × 400 × 148 and a voxel size of 0.68 × 0.68 × 1.5 mm^3^. In the stereo and quantitative analysis of FDG‐PET, standardized test results using *z*‐score were visualized by three‐dimensional stereotactic surface projection (3D‐SSP). Reference was set as the individual's global brain metabolism. The analysis of the PET images was performed on Cortex ID Suite (GE Healthcare). *Z*‐scores ≥2 or ≤−2 were selected as clinically significant hypermetabolism or hypometabolism, respectively (Patterson, Lilien, Takalkar, & Pinkston, 2011; Probasco et al., 2017). To describe clinical improvement, patients received cognitive assessments based on the Mini‐Mental State Examination (MMSE) (Shyu & Yip, 2001) and Montreal Cognitive Assessment (MoCA) (Tsai et al., 2011) at both the subacute and recovery stages.

### 2.4 Receptor mapping

Map of neuronal surface antigens at the gene expression level was adapted from the Allen Human Brain Atlas, which is an open‐source database of quantitative gene‐level transcriptome from DNA microarrays (Allen Institute for Brain Science. Allen Human Brain Atlas. 2010. Available from: human.brain‐map.org) (Hawrylycz et al., 2012). Map at the protein level was modified from Zilles's protein density map of transmitter receptors, in which the regional protein concentration of multiple transmitter receptors was determined based on postmortem human brain (Palomero‐Gallagher, Amunts, & Zilles, 2015). To visualize the distribution and intensity of individual receptors, the regional mean densities of all cortical layers from the supplementary materials of the paper by Zilles and Palomero‐Gallagher (2017) were projected on a spatial brain atlas based on Brodmann areas. Several cytoarchitectural regions had no direct equation to the areas on Brodmann's cortical map. Therefore, we used anatomical approximation to determine the location and average protein densities of the composite cytoarchitectural regions (Table S1). Spatial projection of the density map was performed on FMRIB Software Library (FSL) (Smith et al., 2004) and was visualized using the MRIcron software program.

## 3 RESULTS

### 3.1 Clinical scenario and FDG‐PET findings

**Figure 1:**
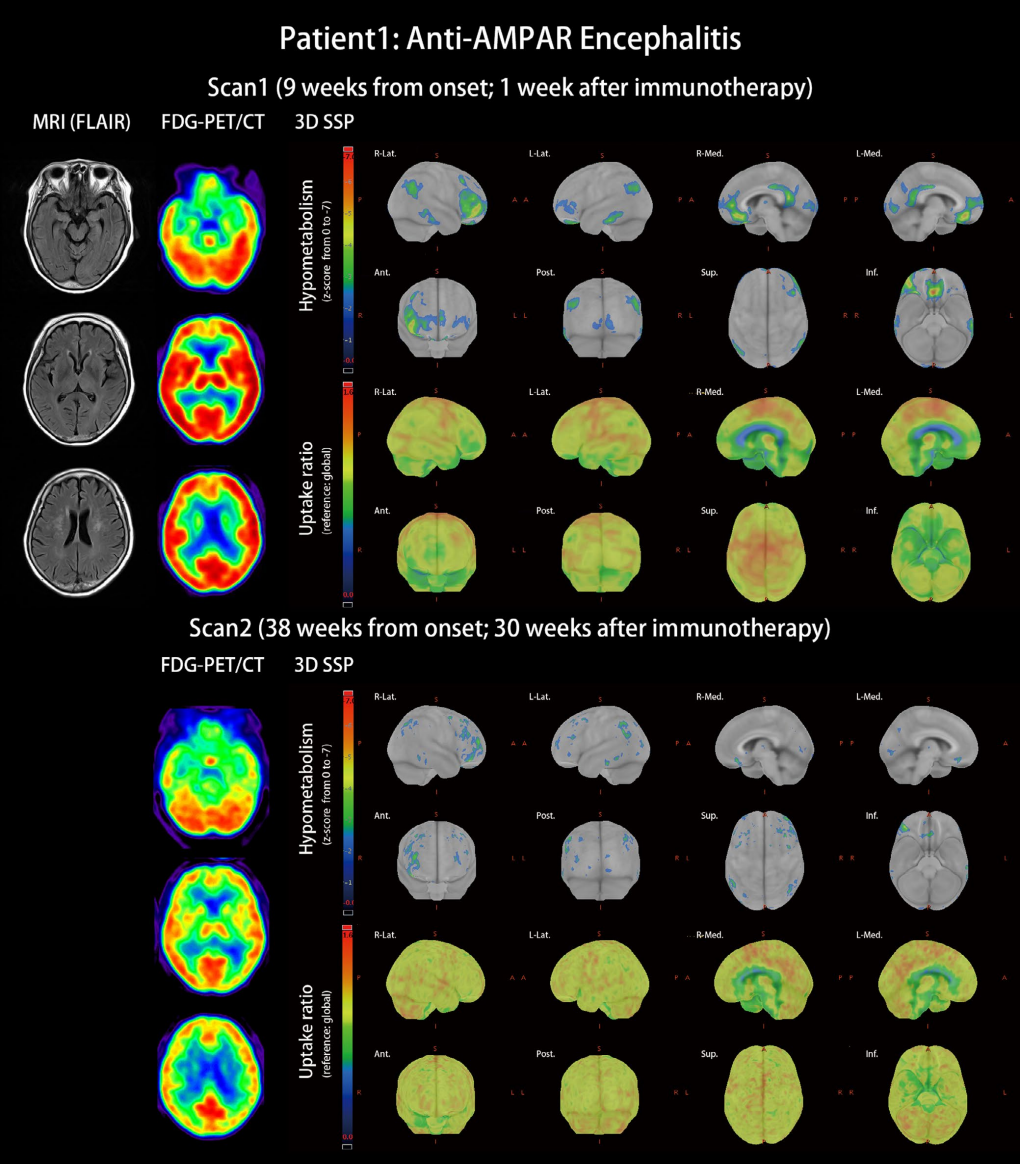
MRI and FDG‐PET/CT of anti‐AMPAR encephalitis (patient 1). Patient 1 was a 66‐year‐old female patient presenting with acute psychosis, personality change, progressive aphasia, and consciousness impairment 3 days after surgery to remove left breast cancer. The diagnosis of anti‐AMPAR encephalitis was made based on clinical symptoms and antibodies detected in her CSF and serum. Initial MRI was nonspecific, but brain FDG‐PET/CT was widely affected with right frontal‐dominant hypometabolism as well as posterior parietal, lateral temporal, and primary visual cortex hypometabolism. Primary sensorimotor cortices were relatively more active than other cortical areas. After immunotherapy, improvement of cortical hypometabolism accompanied clinical improvement. On the second PET scan, only scattered hypometabolism was noticed over the previously affected areas

Patient 1 with anti‐AMPAR encephalitis: This was a 66‐year‐old woman with type 2 diabetes mellitus. She was diagnosed as having left breast invasive ductal carcinoma and was scheduled to have mastectomy surgery. The surgery was successful; pathologist reported the cancer as invasive ductal carcinoma, grade 1, pT2, ER (+), PR(+), Her2(−). However, ataxic gait and acute psychosis with auditory hallucination, agitation, uncontrolled rage, progressive aphasia, anterograde amnesia, confusion, and disorientation developed 3 days after cancer surgery. Infectious and metabolic etiologies were excluded from complete surveillances. MRI of the brain revealed only few spots of white matter hyperintensities. EEG did not find epileptiform discharge. The cell‐based assay found anti‐AMPAR antibodies in her CSF and serum. She was diagnosed as having anti‐AMPAR encephalitis and started immunotherapy at the 8th week after the symptom onset. FDG‐PET/CT at the 9th week indicated extensive hypometabolic areas including the prefrontal, orbitofrontal, temporal parahippocampal, and posterior parietal areas (Figure 1, scan 1). The patient received methylprednisolone pulse therapy by 1 mg/day, followed by oral prednisolone 0.5–1 mg/kg. She also completed 12 sessions of plasmapheresis. Amantadine 200 mg/day as a NMDAR antagonist was used to control limb unsteadiness. Quetiapine 75 mg/day controlled her psychosis. Her consciousness and ataxia gradually improved. Six months later, the patient was independent (mRS score 1) with only mild ataxic gait and mild difficulties in the comprehension of complex sentences remained. The follow‐up FDG‐PET demonstrated that the hypometabolism was resolved (Figure 1, scan 2). Compared with the cognitive performance at the time of the first PET scan, the scores in the second PET scan were significantly improved; MMSE improved from 12 to 24 points and MoCA from 4 to 18 points. The years of school education of this patient was 3 years.

**Figure 2:**
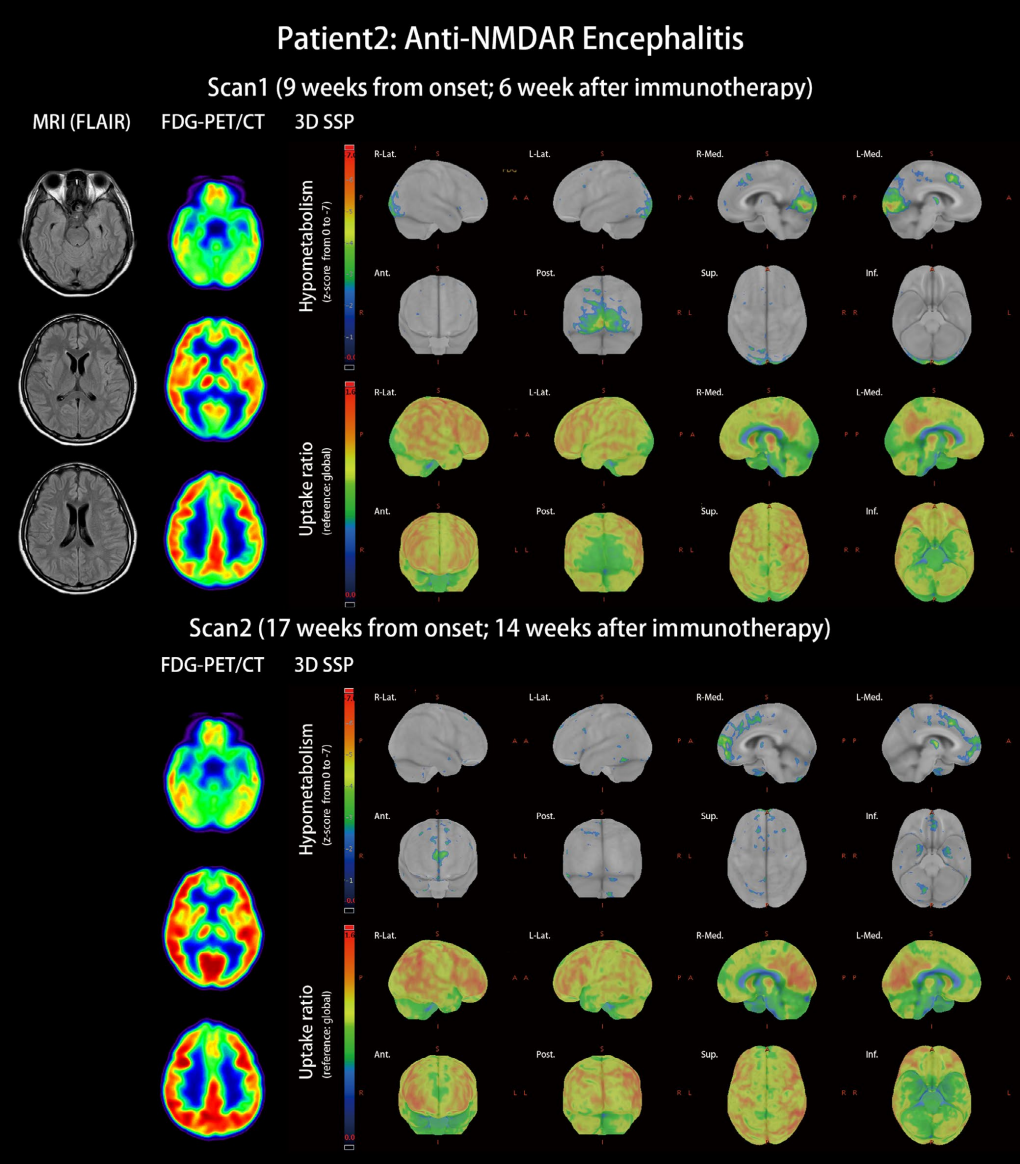
MRI and FDG‐PET/CT of anti‐NMDAR encephalitis (patient 2). A 29‐year‐old female patient with subacute psychosis, consciousness alternation, and status epilepticus for 3 weeks was diagnosed as having anti‐NMDAR encephalitis. T2‐weighted FLAIR images of MRI revealed mild edema and hyperintensities of the bilateral mesial temporal area. After 6 weeks of immunotherapy, FDG‐PET/CT at the 9th week from the first symptoms revealed occipital‐dominant hypometabolism, which was compatible with previously reported distinct hypometabolic patterns of anti‐NMDAR encephalitis. Relative hypermetabolism at the primary sensorimotor cortices, lateral temporal area, inferior parietal area, and pons was noticed in the uptake ratio of scan 1. The occipital hypometabolism resolved in the second PET scan at the 17th week from onset, but new medial frontal to anterior cingulate hypometabolism occurred

Patient 2 with anti‐NMDAR encephalitis: This was a 29‐year‐old female patient without underlying disease. She experienced subacute onset apathy, aphasia that progressed to mutism, amnesia, psychosis, dyskinesia, consciousness change, and status epilepticus. Antibodies against NMDAR were detected in the patient's serum, plasma, and CSF. Under the diagnosis of anti‐NMDAR encephalitis without tumor association, she received immunotherapy from the third week after onset with steroid pulse therapy and plasmapheresis. Initial brain MRI was unspecific. After recovering from status epilepticus and regaining consciousness, the patient underwent FDG‐PET/CT scan 9 weeks after onset. The scan revealed occipital hypometabolism including hypometabolism in the primary and associated visual cortices mixed with hypermetabolism at the right lateral temporal and right inferior parietal lobes (Figure 2, scan 1, and Table S2). At that time, cognitive impairment was quantified as 19 points for MMSE and 12 points for MoCA under the background of 18 years of school education. Her symptoms of anti‐NMDAR encephalitis kept improving under immunotherapy, including plasma exchange, oral prednisolone, azathioprine, and rituximab. Two months after the first FDG‐PET/CT scan, her mRS score was 2, and her cognitive performance was 26 points for MMSE and 27 points for MoCA. The second PET scan exhibited some improvement for occipital hypometabolism but revealed new hypometabolism in the bilateral medial frontal and right anterior cingulate gyrus (Figure 2, scan 2).

### 3.2 Gene expression of NMDAR and AMPAR

**Figure 3:**
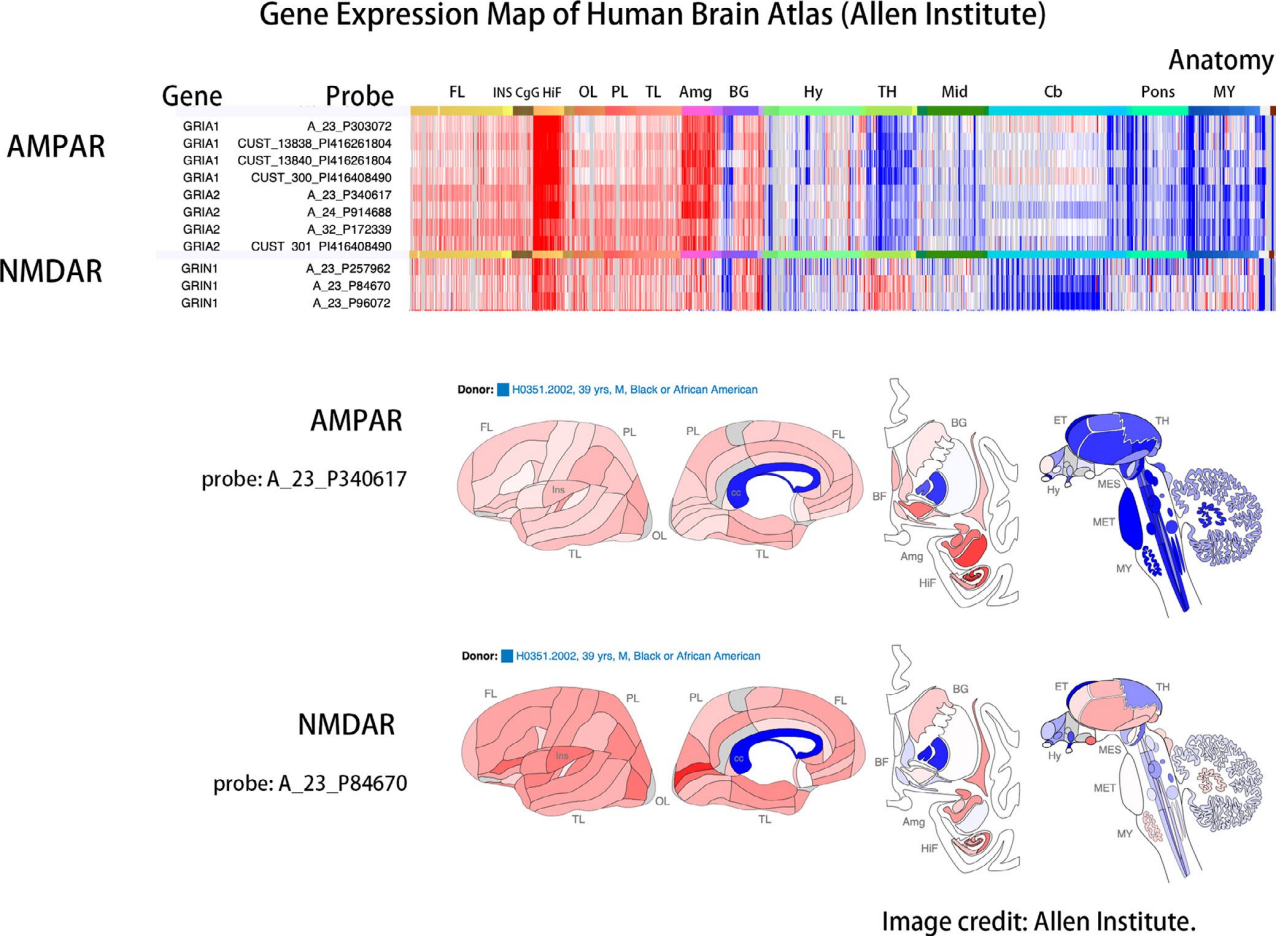
Gene expression level of the Human Brain Atlas of Allen Institute. Genetic expression levels varied according to brain regions. Allen's Human Brain Atlas (available from: human.brain‐map.org) provided microarray expression by anatomical segmentations. Through various probes, the patterns of genetic expression were similar with some differences between AMPAR and NMDAR. A high hippocampal level of gene expression was observed in AMPAR and NMDAR. Expression of the thalamus was noticed in NMDAR but not AMPAR. Genetic expression was neutral in the cerebellum for AMPAR but low for NMDAR. The pontine and medullar expression level was low for AMPAR but moderate for NMDAR. Abbreviations: FL, frontal lobe. INS, insula. CgG, cingulate gyrus. HiF, hippocampal formation. OL, occipital lobe. PL, parietal lobe. TL, temporal lobe. Amg, amygdala. BG, basal ganglia. Hy, hypothalamus. TH, thalamus. Mid, midbrain. Cb, cerebellum. Pons, pons. MY, myelencephalon

Data from the open‐source database of the Allen Institute, the Allen Human Brain Atlas, revealed genetic differences between brain regions (Figure 3). Each gene was detected through several probes to enable variations between tests. From the perspective of whole‐brain gene expression, AMPAR and NMDAR were expressed abundantly in the hippocampal formation, and AMPAR was also rich in the amygdala. AMPAR and NMDAR also had moderate expression in the basal ganglia. In the cerebellum, gene expression was low for NMDAR but neutral for AMPAR. In the pons and medulla, AMPAR was not expressed much, but NMDAR was moderately expressed.

### 3.3 Protein density of NMDAR and AMPAR

**Figure 4:**
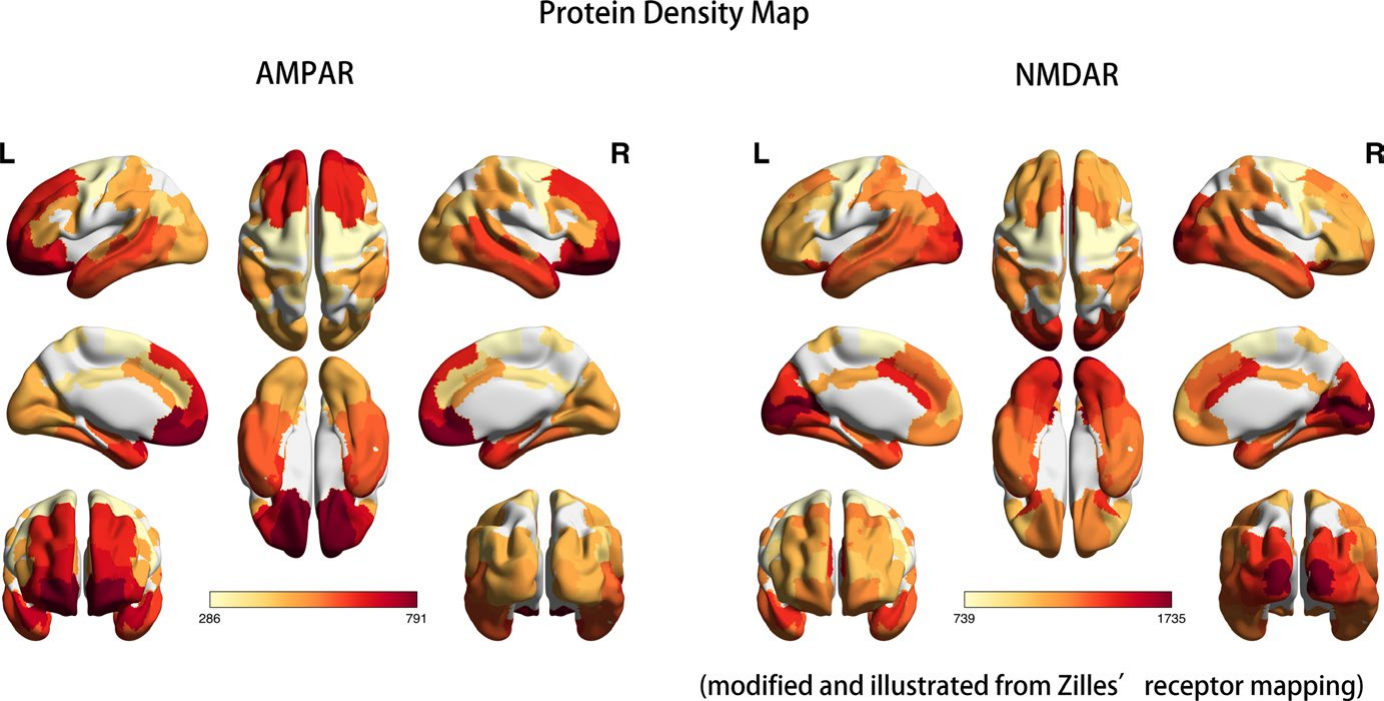
Protein density map modified from Zilles's receptor map. Zilles et al published the cytoarchitecture features of brain regions in 2017 (Zilles & Palomero‐Gallagher, 2017). They stated that the amount and layer difference of each transmitter receptor defined regions of the brain. AMPAR and NMDAR are both glutaminergic receptors of the neuronal surface. We transformed Zilles's brain regions to Brodmann areas and averaged the protein densities of the original regions to Brodmann areas. When comparing their distributions and densities, temporal and parietal association areas were both moderately dense for AMPAR and NMDAR; however, the frontal density was high for AMPAR, and the occipital density was high for NMDAR. Both AMPAR and NMDAR spared the primary motor, primary sensory, and primary auditory cortices. The unit of protein density was fmol/mg of protein

Figure 4 presents three‐dimensional visualization of protein densities with heat map labeling; the red area refers to relatively high protein densities, and the light‐yellow area represents low protein densities. Both AMPAR and NMDAR are glutamatergic receptors that work together in maintaining the excitatory postsynaptic transmission of action potentials. In contrast to the high protein density of the frontal lobe of AMPAR, NMDAR density was higher in the occipital lobe including the primary visual cortex and associated visual cortices. The frontal‐to‐occipital gradient of AMPAR and the occipital‐to‐frontal gradient were opposite in direction. In addition, both AMPAR and NMDAR were richly expressed in the temporal area including both the medial and lateral temporal lobes. Parietal association cortices also contained moderate amounts of AMPAR and NMDAR.

### 3.4 Comparison of receptor map and hypometabolic distribution

In Zilles's receptor map, the occipital lobe was rich in NMDAR but poor in AMPAR, and the frontal lobe was abundant in AMPAR but relatively lacking in NMDAR (Figure 4). The frontal‐predominant hypometabolism of anti‐AMPAR encephalitis (Figure 1) and the occipital‐predominant hypometabolism of anti‐NMDAR encephalitis (Figure 2) reflected the opposite frontal–occipital protein density gradients of AMPAR and NMDAR (Figure 4). However, the frontal–occipital gradients of AMPAR and NMDAR were nonsignificant on Allen's gene expression map (Figure 3). Therefore, the topographic variation of functional impairment by FDG‐PET hypometabolism was best correlated to the locations of high protein density.

## 4 DISCUSSION

### 4.1 FDG‐PET and its clinical implications in autoimmune encephalitis

Because of the high brain background activity, FDG‐PET/CT is conventionally considered less accurate than MRI in identifying encephalitis (Tunkel et al., 2008). However, FDG‐PET/CT may facilitate the diagnosis of autoimmune encephalitis (Quartuccio et al., 2015). In autoimmune encephalitis, the cortical hypometabolism may or may not be combined with hypermetabolic foci (Fisher, Patel, Lai, & Schulz, 2012). *Z*‐score and 3D‐SSP visualization help clinical physicians explain patients' cognitive dysfunction in correlation with regions of glucose hypometabolism and are applicable to clinical use (Solnes et al., 2017). The receptor maps of gene expression and protein density helped us understand the topographical distribution and correlations with FDG‐PET hypometabolism regions.

FDG‐PET detected anomalies in autoimmune encephalitis more frequently than did of electroencephalography, MRI, and CSF. Regional brain hypometabolism (69%) was more frequently detected than isolated hypermetabolism (3%) or mixed hyper‐ and hypometabolism (13%) (Probasco et al., 2017). The functioning of the brain depends heavily on glucose use (Mergenthaler, Lindauer, Dienel, & Meisel, 2013); neuronal activities drive local glucose metabolism through direct glucose uptake by neurons (Lundgaard et al., 2015), astrocyte–neuron interaction, oxidative energy transmission, and increase of regional cerebral blood flow (Magistretti & Allaman, 2015). Focal hypometabolism in brain FDG‐PET represents lowered local neuronal activities, which result in regional brain dysfunction and clinical symptoms. The mechanism of the decrease of glucose metabolism in autoimmune encephalitis could be cortical dysfunction mediated by antibodies; anti‐NMDAR antibodies cause receptor internalization (Moscato et al., 2014), synaptic glutamatergic dysfunction (Manto, Dalmau, Didelot, Rogemond, & Honnorat, 2010), decrease of synaptic NMDAR‐mediated currents (Hughes et al., 2010), and suppression of synaptic plasticity (Zhang et al., 2012). Similar pathogenic mechanisms were observed in antibodies against AMPAR, which induce receptor internalization, receptor reorganization, decrease of AMPAR‐mediated excitatory postsynaptic potential, and impaired synaptic plasticity (Haselmann et al., 2018; Peng et al., 2014). By impairing these functioning proteins on the neuronal surface, pathogenic antibodies reduce normal neuronal activities, produce cognitive and psychiatric symptoms, and make cortical hypometabolism appear on brain FDG‐PET. We proposed the patterns of cortical hypometabolism also differentiate certain types of autoimmune encephalitis.

### 4.2 Brain glucose metabolic patterns in autoimmune encephalitis

To date, several understandings of characteristic metabolic patterns of autoimmune encephalitis have been published. Early hypermetabolic lesion in the mesial temporal areas could be a marker of active inflammatory process of limbic encephalitis (Probasco et al., 2017), because FDG usually accumulates at infection or inflammation foci (Bleeker‐Rovers, de Kleijn, Corstens, van der Meer, & Oyen, 2004). Occipital hypometabolism is characteristic in patients with anti‐NMDAR encephalitis (Probasco et al., 2018). Seizure commonly occurs in autoimmune encephalitis and could last long during disease course (Spatola, Stojanova, Prior, Dalmau, & Rossetti, 2014); the influence of epileptic activities should be considered, that is, hypometabolism in epileptogenic foci but hypermetabolic area extended beyond the seizure onset zone (la Fougere, Rominger, Forster, Geisler, & Bartenstein, 2009; Nelissen et al., 2006). Based on our case study and literature review, several components of FDG‐PET metabolic disorders characterizing autoimmune encephalitis were summarized thereafter.

#### 4.2.1 Temporal hypermetabolism

Temporal hypermetabolism, especially in the mesial temporal area, appeared early in autoimmune encephalitis (Baumgartner et al., 2013). An increase of FDG uptake in the mesiotemporal area could even start before symptoms of encephalitis. In a case series of anti‐NMDAR encephalitis, temporal hypermetabolism was noticed in a patient at the presymptomatic stage (Leypoldt et al., 2012). In anti‐LGI1 encephalitis, medial temporal hypermetabolism indicated a more severe condition, whereas the absence of medial temporal hypermetabolism was related to good outcome (Shin et al., 2013). Similarly, mesial temporal lobe hyperintensity in T2‐weighted images of brain MRI was characteristic of autoimmune limbic encephalitis. The correlation between PET and MRI could be explained by high antibody affinity around the hippocampus, parahippocampal area, and amygdala (Bauer & Bien, 2016).

#### 4.2.2 Frontal hypermetabolism in anti‐NMDAR encephalitis

A previous case study suggested that frontal hypermetabolism in anti‐NMDAR encephalitis represented altered uneven brain metabolism with frontal–occipital gradient (Leypoldt et al., 2012). Because *z*‐scoring was a relative but not absolute value of brain metabolism, regional hypermetabolism and hypometabolism indicated regional differences of disease process. Ketamine, a noncompetitive NMDAR open‐channel blocker, affects human brain to simulate anti‐NMDAR antibodies' receptor blocking effects. Ketamine acts on NMDAR and results in NMDAR containing GABAergic interneuron hypofunction and subsequent pyramidal neuron hyperexcitability in the frontal cortex (Autry et al., 2011; Li et al., 2010; Nosyreva et al., 2013). MK‐801, another noncompetitive NMDAR antagonist, induced hyperlocomotion by elevating the frontal neuronal activity in the orbitofrontal area of mice (Seiriki et al., 2016). Schizophrenia was also mediated by frontal GABAergic activity through reduced NMDAR function (Cohen, Tsien, Goff, & Halassa, 2015). Hypermotor symptoms, including complex limb movement and orolingual dyskinesia, and psychosis with hallucinations are common symptoms in anti‐NMDAR encephalitis. Therefore, anti‐NMDAR antibodies, ketamine, MK‐801, and the pathological process of schizophrenia all cause NMDAR hypofunction and result in symptoms such as hallucination and hypermotor movement disorders.

#### 4.2.3 Occipital hypometabolism in anti‐NMDAR encephalitis

Currently, the most consistent metabolic pattern that represents neuronal dysfunction in autoimmune encephalitis is the occipital hypometabolism in anti‐NMDAR encephalitis (Maeder‐Ingvar et al., 2011). Because anti‐NMDAR encephalitis has a relatively higher incidence compared with other autoimmune encephalitis, accumulated evidence is sufficiently abundant to conclude this distinct pattern, its relatively high prevalence in severe cases, and its reversibility in recovery (Leypoldt et al., 2012; Probasco et al., 2018). However, the reason why the occipital lobe is frequently involved was not explained. In our study, we introduced cytoarchitecture concepts to understand the metabolic unbalance in autoimmune encephalitis. As illustrated in Figures 3 and 4, genetic expression patterns were similar between AMPAR and NMDAR; the protein densities of AMPAR and NMDAR were both high in the temporal and parietal multimodal association areas, but the densities were more frontal‐dominant for AMPAR and more occipital‐dominant for NMDAR. The high occipital density of NMDAR echoes the occipital hypometabolism in our patient and other case series (Leypoldt et al., 2012; Probasco et al., 2018; Solnes et al., 2017; Yuan et al., 2016).

#### 4.2.4 Hypometabolism in association cortices

Multimodal association areas have wide connections to other brain areas and own their specific cytoarchitecture fingerprints that can separate areas and identify their hierarchical position (Zilles & Palomero‐Gallagher, 2017). Hypometabolism in association cortices has been reported as a frequent FDG‐PET finding in patients with autoimmune encephalitic (Baumgartner et al., 2013). In our study, the involvement of the prefrontal association cortex, temporal parahippocampal association cortex, and parietal association cortex was observed in patients with anti‐AMPAR encephalitis (Figure 1). During evolution, these multimodal association cortices evolved to enlarge in human (Rakic, 2009) and work together to form cognition. Information from primary cortices flows into multimodal association cortices to create a higher level of function. The prefrontal association cortex manages executive function, which if defected leads to difficulties of planning (Purves et al., 2004); the temporal parahippocampal association cortex reserves information from all sensory modalities and controls emotion and recognition, which if damaged leads to prosopagnosia, episodic memory deficits, and impaired spatial memory (Aminoff, Kveraga, & Bar, 2013; Ploner et al., 2000); the perisylvian association cortex serves for language integration (Catani, Jones, & Ffytche, 2005), which if lesioned causes aphasia and impaired verbal memory; the parietal association cortex controls attention and salience by monitoring the auditory and visual sensory inputs from primary cortices, which if injured impacts goal‐directed behavior (Cohen, 2009) and contralateral neglect syndrome (Purves et al., 2004). These symptoms of neuronal dysfunction of association cortices are common in autoimmune encephalitis (Dalmau & Graus, 2018).

#### 4.2.5 Sparing of unimodal primary motor cortex

Primary cortices were usually not affected by autoimmune encephalitis (Baumgartner et al., 2013). In a case series, the sparing of the primary motor cortex occurred in half of the abnormal FDG‐PET scans of limbic encephalitis (Masangkay, Basu, Moghbel, Kwee, & Alavi, 2014). Bilateral motor cortex sparing was also observed in the FDG‐PET of a case of anti‐AMPAR encephalitis (Laurido‐Soto et al., 2019). In our patients, cortical hypometabolism also did not include primary motor cortex.

#### 4.2.6 Reversibility in recovery

In this study, the patients exhibited clinical and imaging improvement after immunotherapy (Figures 1 and 2). The reversibility of clinical symptoms has been linked to reversible receptor function after removing the antibodies from receptors (Dalmau, Lancaster, Martinez‐Hernandez, Rosenfeld, & Balice‐Gordon, 2011; Hughes et al., 2010; Lai et al., 2009; Zhang et al., 2012). In our case study, clinical recovery also correlated to the resolution of metabolic unbalance in FDG‐PET. Regional low glucose metabolism represented regional brain dysfunction; normalization of brain glucose metabolism occurred concurrently with the recovery of cognitive function. Therefore, the clinical reversibility of cognition, functional reversibility of receptors, and imaging reversibility of FDG‐PET all depend on antibody concentration.

### 4.3 Considerations of the study

In view of the low incidence of autoimmune encephalitis, especially anti‐AMPAR encephalitis, large number of cases is difficult to accumulate. Although we reported only two cases, the collection of serial brain FDG‐PET functional imaging and complete cognitive assessments are still worth reporting. Second, FDG‐PET scan required cooperative patients and preferably free from sedative medication. Therefore, PET imaging was usually taken when patients were recovering from a severe condition and therefore were not able to represent the most striking influences of antibodies on brain. In this case report, FDG‐PET/CT was arranged as early as patients' condition allowed them to take the examination to estimate disease‐related cortical dysfunction. Third, the reference of FDG‐PET analysis was set as the individual's global brain metabolism. Self‐reference carried the possible bias especially when individuals' brain metabolism globally decreased. Setting up a group of age‐matched normal controls as reference could eliminate the bias. Although currently not available for this study, the normal control database will help to improve the quality of future work. Fourth, the receptor maps at the gene expression and protein levels were based on donors of dissimilar age, gender, and ethnicity. We also noticed individual variation of gene expression in the Allen Human Brain Atlas. Projecting the receptor map onto patient's brains might have represented bias for interpersonal differences of protein/gene expression and variations of spatial alignment. Finally, although Zilles's segmentation originated from Brodmann areas, it was partly beyond Brodmann (Amunts & Zilles, 2015); transforming Zilles's cytoarchitecture segmentation into Brodmann areas potentially carried bias during the processing.

## 5 CONCLUSIONS

FDG‐PET/CT has a diagnostic role in autoimmune encephalitis, being characterized by the hypometabolic areas, in representing receptor hypofunction and topographical distribution of the antigenic receptor. The metabolic disturbance of brain in autoimmune encephalitis is reversible after treatment, in corresponding to cognitive improvement. Therefore, FDG‐PET is a functional indicator for disease progression and response to treatment in autoimmune encephalitis. In literature review, we summarize six points of characteristics of brain FDG‐PET in autoimmune encephalitis that were (a) temporal hypermetabolism, (b) frontal hypermetabolism and (c) occipital hypometabolism in anti‐NMDAR encephalitis, (d) hypometabolism in association cortices, (e) sparing of unimodal primary motor cortex, and (f) reversibility in recovery.

## CONFLICT OF INTEREST

All authors report no conflicts of interest.

## Data Availability

The data that support the findings of this study are available from the corresponding author upon reasonable request.
