# Supplementary material for: Different FDG‐PET metabolic patterns of anti‐AMPAR and anti‐NMDAR encephalitis: Case report and literature review
Source: Brain Behav. 2020 Jan 27;10(3):e01540. doi: 10.1002/brb3.1540 (PMC7066351; doi:10.1002/brb3.1540)
Supplement: Supplementary file 1 [file BRB3-10-e01540-s001.docx]

**Table S1 Transformation of Zilles’ receptor densities to Brodmann areas**

| Zilles’ Areas | Corresponding Brodmann Areas in illustration | AMPAR Mean Densities | NMDAR Mean Densities |
| --- | --- | --- | --- |
| 1 | 1 | 443 | 1169 |
| 2 | 2 | 478 | 1271 |
| 3a, 3b | 3 | 464 | 1216 |
| 4 | 4 | 286 | 739 |
| 5L, 5M | 5 | 374 | 1047.5 |
| 6 | 6 | 301 | 751 |
| 8 | 8 | 654 | 1255 |
| 9 | 9 | 647 | 1123 |
| 10L, 10M | 10 | 688.5 | 1042.5 |
| 11 | 11 | 791 | 1234 |
| V1 | 17 | 496 | 1735 |
| V2d, V2v | 18 | 461.5 | 1449 |
| V3A, V3d, V3v, V4v | 19 | 468 | 1407.75 |
| 20 | 20 | 584 | 1257 |
| 21 | 21 | 618 | 1314 |
| 22 | 22 | 506 | 1212 |
| 23 | 23 | 364 | 1137 |
| 24 | 24 | 509 | 1478 |
| 31 | 31 | 400 | 1234 |
| 32 | 32 | 383 | 1299 |
| 36 | 36 | 638 | 1343 |
| FG1, FG2, 37B, 37L, 37M | 37 | 566.2 | 1320.6 |
| 38 | 38 | 715 | 1432 |
| PGa, PGp | 39 | 399 | 1200.5 |
| PFt, PFm | 40 | 499 | 1203 |
| 41 | 41 | 479 | 1266 |
| 42 | 42 | 498 | 1272 |
| 44 | 44 | 492 | 964 |
| 45 | 45 | 472 | 1078 |
| 46 | 46 | 644 | 1091 |
| 47 | 47 | 689 | 1033 |

Mean densities of receptor were expressed in fmol/mg of protein.

**Table S2 Z-scores of FDG-PET metabolism**

| Type of Antibody | | | AMPAR | | NMDAR | |
| --- | --- | --- | --- | --- | --- | --- |
| Scan | | | Scan1 | Scan2 | Scan1 | Scan2 |
| Time from onset (week) | | | 9 | 38 | 9 | 17 |
| Time from treatment (week) | | | 1 | 30 | 6 | 14 |
| Reference | | | Global | Global | Global | Global |
| Prefrontal | Lateral | R | -3.79 | -3.43 | 0.26 | 0.20 |
|  |  | L | -1.31 | -1.87 | -0.40 | -1.17 |
|  | Medial | R | 0.49 | 1.44 | -0.19 | -3.78 |
|  |  | L | 0.83 | 1.57 | -0.64 | -3.93 |
| Sensorimotor |  | R | 3.71 | 0.55 | 2.75 | 0.64 |
|  |  | L | 4.50 | 1.05 | 0.82 | 0.35 |
| Anterior cingulate |  | R | -1.39 | 1.34 | -0.50 | -2.23 |
|  |  | L | -1.04 | 1.08 | 0.00 | -1.58 |
| Posterior cingulate |  | R | -2.44 | -0.49 | -0.06 | -0.57 |
|  |  | L | -1.85 | -0.53 | -1.04 | -1.03 |
| Precuneus |  | R | -1.11 | -0.74 | -1.41 | 0.63 |
|  |  | L | -0.83 | -0.75 | -2.78 | -0.86 |
| Parietal | Superior | R | 1.77 | 0.46 | 1.69 | 1.06 |
|  |  | L | 3.33 | 0.94 | -0.58 | -0.67 |
|  | Inferior | R | -1.38 | -1.69 | 2.83 | 3.73 |
|  |  | L | -0.13 | -2.01 | -0.38 | -0.53 |
| Occipital | Lateral | R | -1.39 | -1.53 | -4.14 | 1.19 |
|  |  | L | -0.51 | -1.69 | -4.33 | 0.24 |
|  | Primary visual | R | -2.43 | -1.53 | -5.77 | 0.21 |
|  |  | L | -2.29 | -1.78 | -5.20 | 0.42 |
| Temporal | Lateral | R | -0.38 | 1.45 | 2.47 | 2.09 |
|  |  | L | 0.77 | 1.36 | 0.85 | -0.45 |
|  | Mesial | R | -0.58 | 2.79 | 1.27 | -0.83 |
|  |  | L | 0.60 | 4.02 | 0.78 | -1.14 |
| Cerebellum |  |  | 0.50 | 2.84 | 0.58 | -0.57 |
| Pons |  |  | 2.26 | 3.65 | 2.38 | 0.76 |
